# Supplementary material for: Recent Advancements in Polyphenylsulfone Membrane Modification Methods for Separation Applications
Source: Membranes (Basel). 2022 Feb 21;12(2):247. doi: 10.3390/membranes12020247 (PMC8876851; doi:10.3390/membranes12020247)
Supplement: Supplementary file 1 [file membranes-12-00247-s001.zip › membranes-1592640-supplementary.pdf]

membranes

MDPI

Supplementary Materials

Recent Advancements in Polyphenylsulfone Membrane Modification Methods for Separation Applications

Arun Kumar Shukla [1], Javed Alam [1,*] and Mansour Alhoshan [1,2,3,*]

[1] King Abdullah Institute for Nanotechnology, King Saud University, P.O. Box 2455, Riyadh 11451, Saudi Arabia; ashukla@ksu.edu.sa
[2] Department of Chemical Engineering, College of Engineering, King Saud University, P.O. Box 2455, Riyadh 11451, Saudi Arabia
[3] K.A. CARE Energy Research and Innovation Center at Riyadh, P.O.Box 2022, Riyadh 11451, Saudi Arabia
* Correspondence: javaalam@ksu.edu.sa (J.A.); mhoshan@ksu.edu.sa (M.A.)

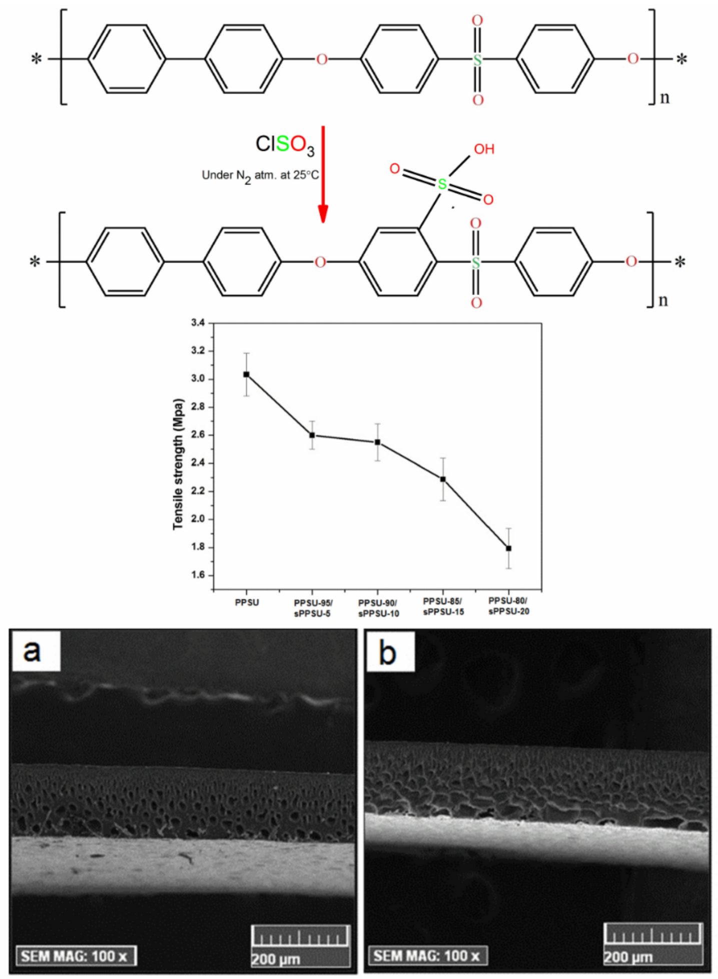

Figure S1. Chemical structure of sulfonation reaction of polyphenylsulfone, prepared membranes mechanical strength, and their cross-sectional morphological images of (a) PPSU (b) sPPSU [1].

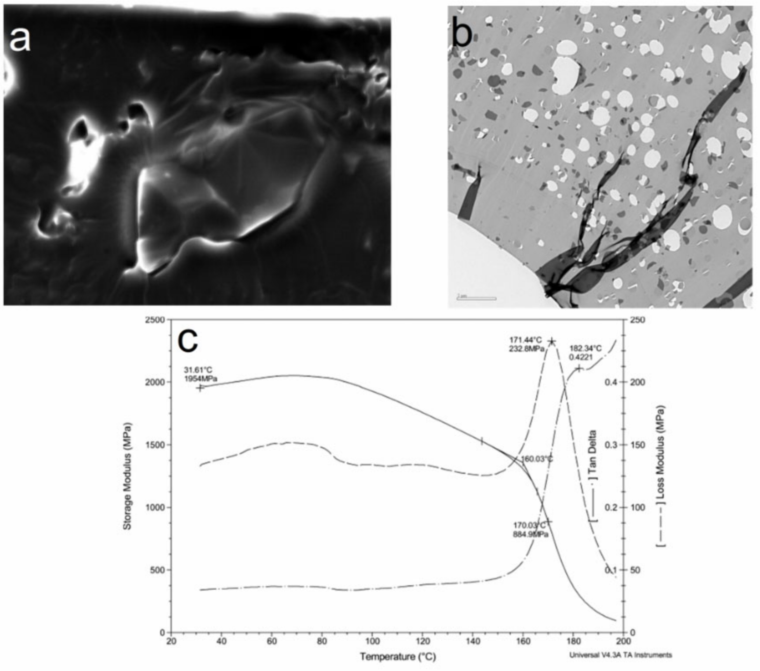

Figure S2. The prepared sPPSU/sPOSS membrane (a) freeze fracture SEM cross section image (b) TEM image, and (c) dynamical mechanical strength properties [2].

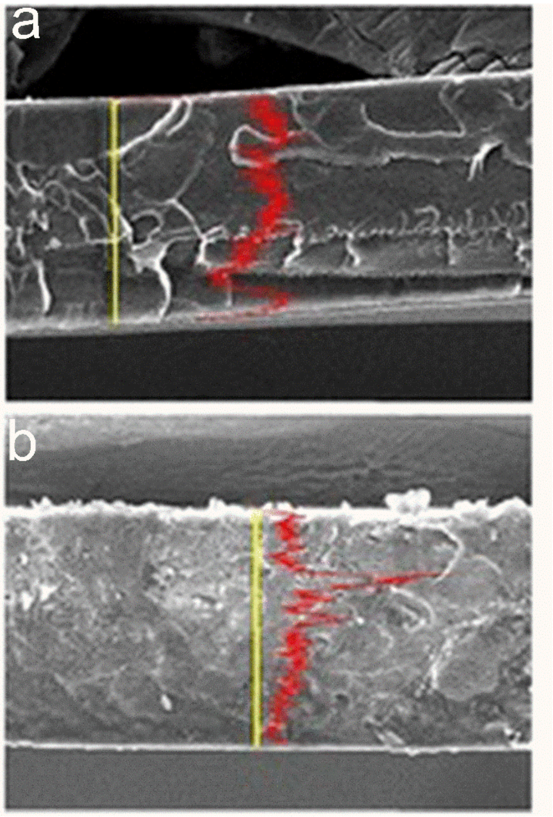

Figure S3. SEM image of membranes for gas separation (a) sPPSU (b) PIM-1/sPPSU [3].

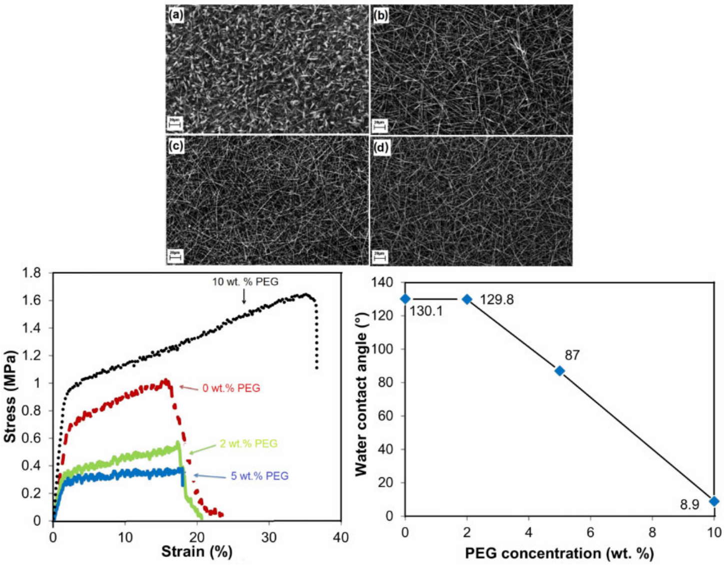

Figure S4. SEM images of the fibrous membranes surfaces with respect to the PPSU with PEG concentration in the electrospinning solution: (a) 0 wt.%, (b) 2 wt.%, (c) 5 wt.%, and (d) 10 wt.%, and mechanical properties and water contact angle [4].

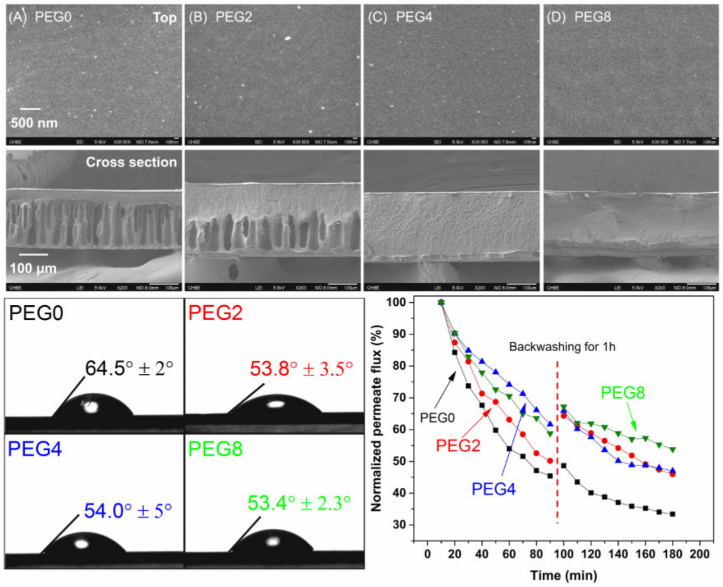

Figure S5. SEM images of the sPPSU membranes surface and cross-section, water contact angles, and filtration performance with PEG concentration [5].

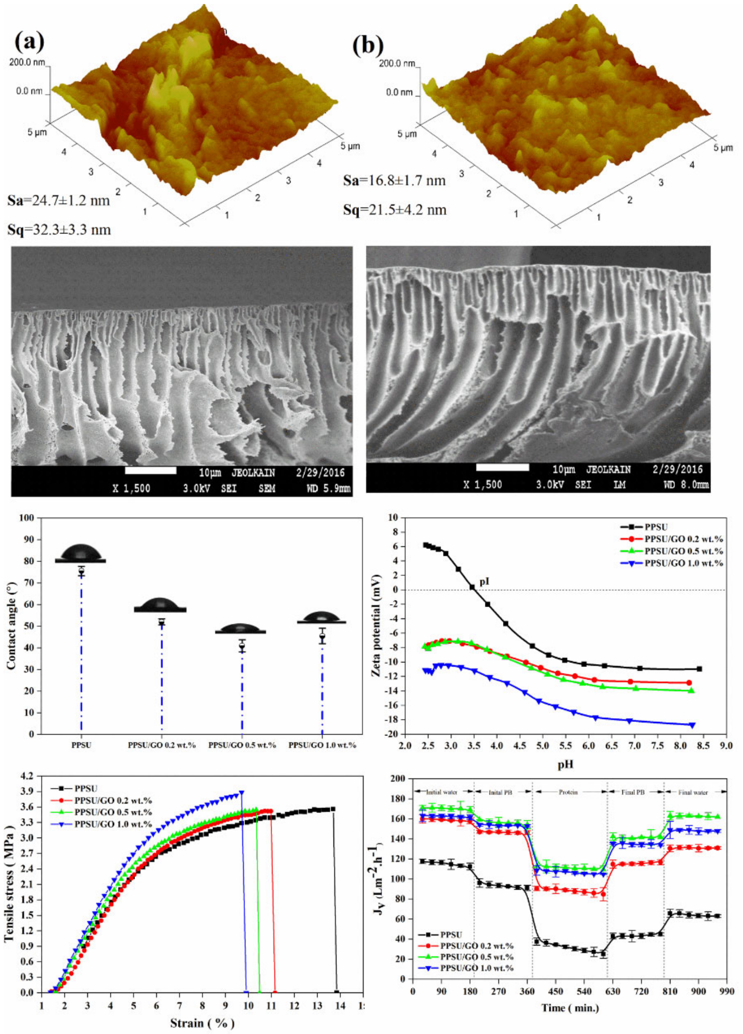

Figure S6. PPSU/GO-based blended membranes (a) AFM and SEM images of PPSU (b) AFM and SEM images of PPSU/GO; and water contact angle, surface zeta potential as a function of pH, mechanical properties using tensile tests, and time-dependent fluxes of pure water, and BSA protein [6].

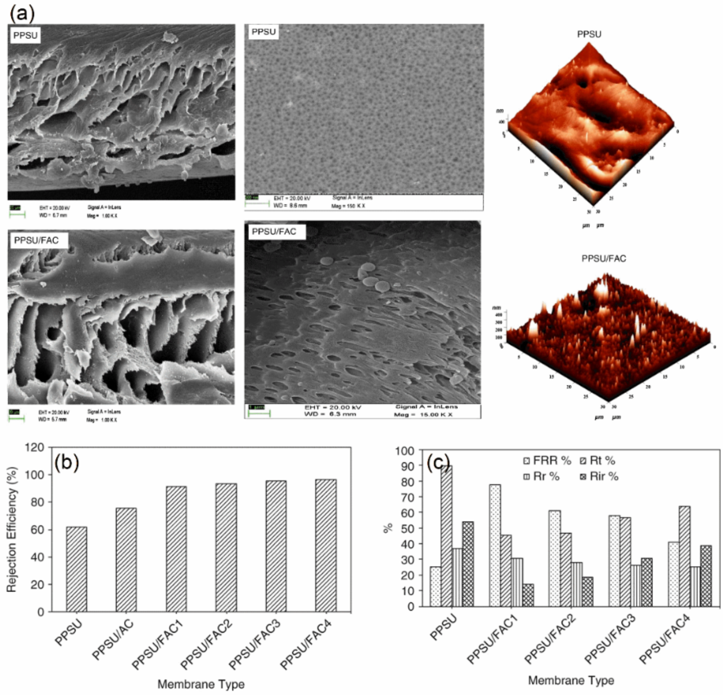

Figure S7. Modified membranes (a) cross section, top surface SEM images and 3D AFM images, (b) rejection efficiency, and (d) flux recovery and fouling resistance ratio [7].

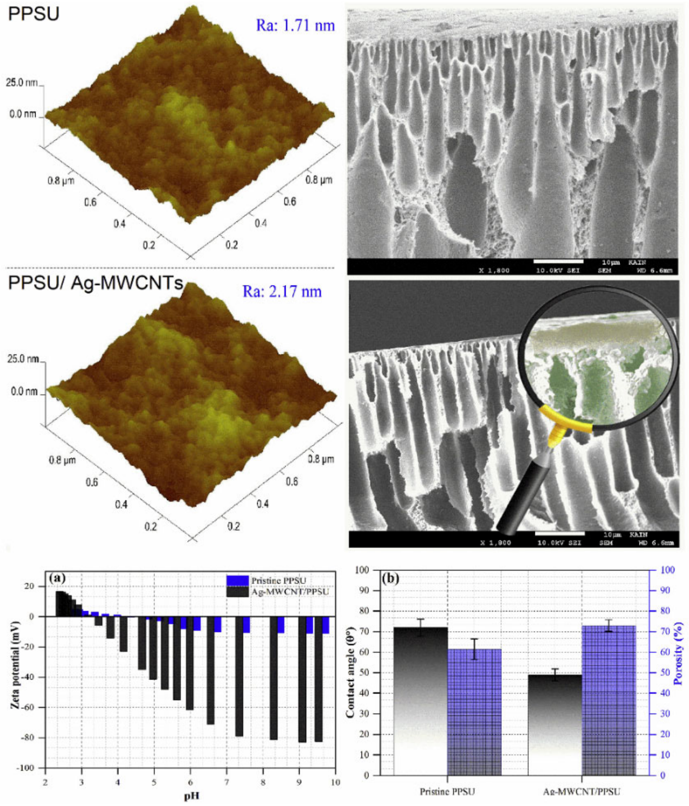

Figure S8. PPSU embedded with Ag-MWCNT membranes: 3D AFM images, low and high magnification SEM images, and (a) zeta potential as a function of pH (b) contact angle and porosity [8].

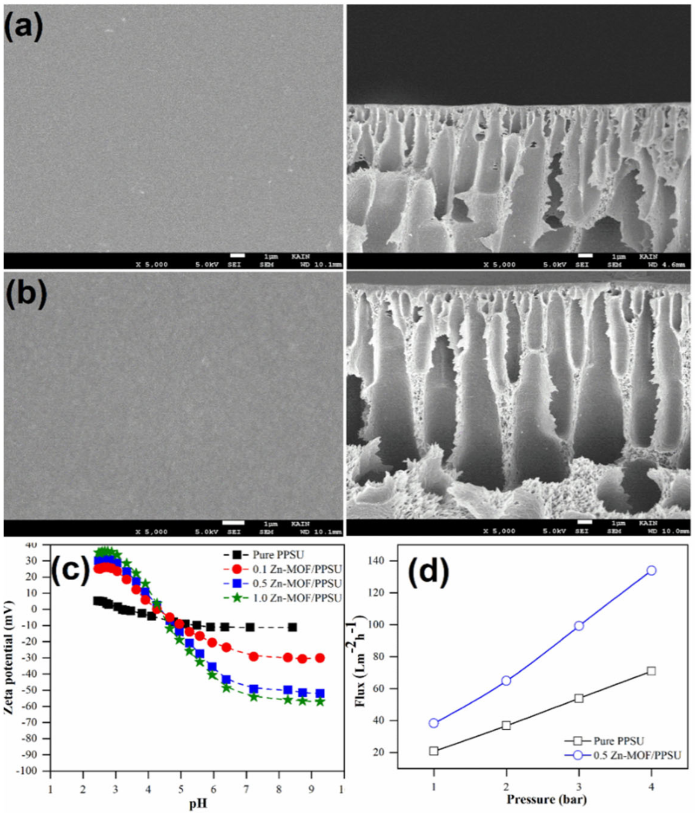

Figure S9. Modified PPSU and PPSU/Zn-MOF membranes: (a and b) surface and cross-section images, (c) zeta potential versus pH, and (d) pure water hydraulic permeability [9].

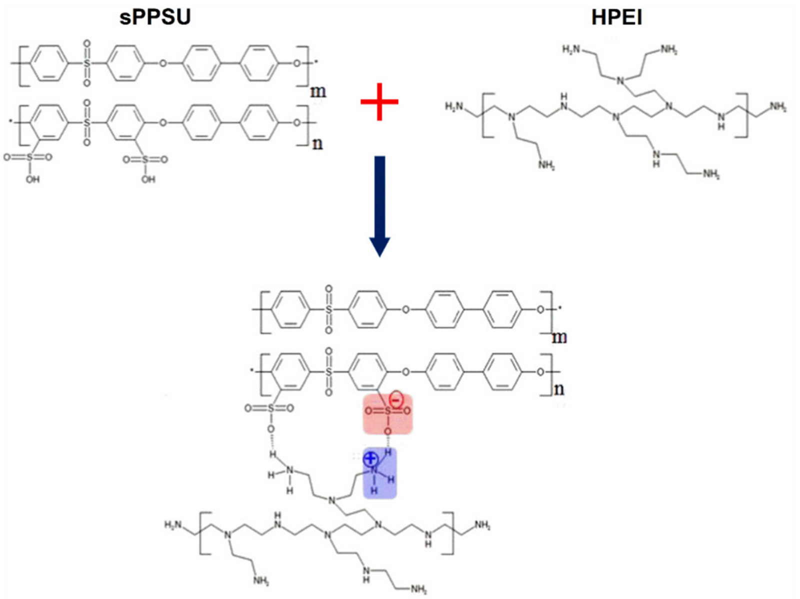

Figure S10. The ionic cross-linking reaction between sPPSU and HPEI at the membrane surface[10].

References

1. Arumugham, T.; Kaleekkal, N.J.; Doraiswamy, M. Development of new hybrid ultrafiltration membranes by entanglement of macromolecular PPSU-SO3H chains: Preparation, morphologies, mechanical strength, and fouling resistant properties. *J. Appl. Polym. Sci.* **2015**, *132*, 1–9.
2. Hartmann-Thompson, C.; Merrington, A.; Carver, P.I.; Keeley, D.L.; Rousseau, J.L.; Hucul, D.; Bruza, K.J.; Thomas, L.S.; Keinath, S.E.; Nowak, R.M.; et al. Proton-conducting polyhedral oligosilsesquioxane nanoadditives for sulfonated polyphenylsulfone hydrogen fuel cell proton exchange membranes. *J. Appl. Polym. Sci.* **2008**, *110*, 958–974.
3. Yong, W.F.; Lee, Z.K.; Chung, T.S. Blends of a polymer of intrinsic microporosity and partially sulfonated polyphenylenesulfone for Gas separation. *ChemSusChem* **2016**, *9*, 1953–1962.
4. Kiani, S.; Mousavi, S.M.; Shahtahmassebi, N.; Saljoughi, E. Hydrophilicity improvement in polyphenylsulfone nanofibrous filtration membranes through addition of polyethylene glycol. *Appl. Surf. Sci.* **2015**, *359*, 252–258.
5. Feng, Y.; Han, G.; Chung, T.S.; Weber, M.; Widjojo, N.; Maletzko, C. Effects of polyethylene glycol on membrane formation and properties of hydrophilic sulfonated polyphenylenesulfone (sPPSU) membranes. *J. Memb. Sci.* **2017**, *531*, 27–35.
6. Shukla, A.K.; Alam, J.; Alhoshan, M.; Dass, L.A.; Muthumareeswaran, M.R. Development of a nanocomposite ultrafiltration membrane based on polyphenylsulfone blended with graphene oxide. *Sci. Rep.* **2017**, *7*, 41976–41987.
7. Saranya, R.; Kumar, M.; Tamilarasan, R.; Ismail, A.F.; Arthanareeswaran, G. Functionalised activated carbon modified polyphenylsulfone composite membranes for adsorption enhanced phenol filtration. *J. Chem. Technol. Biotechnol.* **2016**, *91*, 748–761.
8. Shukla, A.K.; Alam, J.; Ansari, M.A.; Alhoshan, M.; Alam, M.; Kaushik, A. Selective ion removal and antibacterial activity of silver-doped multi-walled carbon nanotube / polyphenylsulfone nanocomposite membranes. *Mater. Chem. Phys.* **2019**, *233*, 102–112.
9. Shukla, A.K.; Alam, J.; Ali, F.A.A.; Alhoshan, M. A highly permeable zinc-based MOF/polyphenylsulfone composite membrane with elevated antifouling properties. *Chem. Commun.* **2020**, *56*, 5231–5234.
10. Asadi Tashvigh, A.; Luo, L.; Chung, T.S.; Weber, M.; Maletzko, C. A novel ionically cross-linked sulfonated polyphenylsulfone (sPPSU) membrane for organic solvent nanofiltration (OSN). *J. Memb. Sci.* **2018**, *545*, 221–228.
